# Paeoniflorin Ameliorates Macrophage Infiltration and Activation by Inhibiting the TLR4 Signaling Pathway in Diabetic Nephropathy

**DOI:** 10.3389/fphar.2019.00566

**Published:** 2019-05-22

**Authors:** Yun-xia Shao, Qian Gong, Xiang-Ming Qi, Kun Wang, Yong-gui Wu

**Affiliations:** ^1^Department of Nephrology, The First Affiliated Hospital, Anhui Medical University Hefei, Hefei, China; ^2^Department of Nephrology, The Second People’s Hospital of Wuhu, Wuhu, China

**Keywords:** paeoniflorin, toll-like receptor 4, macrophages, diabetic nephropathy, inflammation

## Abstract

Paeoniflorin (PF) is the primary component of total glucosides of paeony (TGP). It exerts multiple effects, including immunoregulatory and anti-inflammatory effects. Our previous study has found that PF has a remarkable renal-protective effect in diabetic mice, but exact mechanism has not been clarified. This study mainly explores whether PF affects macrophage infiltration and activation in diabetic kidney through TLR4 pathway. Thus, this study was conducted to investigate the effect of PF on a streptozotocin (STZ)-induced experimental DN model. The results suggested that the onset and clinical symptoms of DN in mice were remarkably ameliorated after the administration of PF. Moreover, the number of infiltrating macrophages in the mouse kidneys was also markedly decreased. Instead of inhibiting the activation of macrophages directly, PF could influence macrophages by suppressing iNOS expression as well as the production of TNF-α, IL-1β, and MCP-1 both *in vivo* and *in vitro*. These effects might be attributable to the inhibition of the TLR4 signaling pathway. The percentage of M1-phenotype cells as well as the mRNA levels of iNOS, TNF-α, IL-1β, and MCP-1 were downregulated when PF-treated polarized macrophages were cultured under conditions of high glucose (HG) levels. In addition, the expression of TLR4, along with that of downstream signaling molecule proteins, was also reduced. Our study has provided new insights into the potential of PF as a promising therapeutic agent for treating DN and has illustrated the underlying mechanism of PF from a new perspective.

## Introduction

Diabetic nephropathy (DN) is a microvascular complication of diabetes. Recent research has found that chronic microinflammation plays a vital role in the pathogenesis of DN (Wada and Makino, 2013). Macrophages play a major regulatory role in the inflammation seen in the renal tissue of early-stage DN patients (Klessens et al., 2017). These macrophages can be activated by the abnormally elevated levels of pathogenic factors, such as glucose, AGEs and renin, in the renal environment. After activation, they can secrete inflammatory cytokines or mediators and chemokines and produce ROS (Tesch, 2010; Sasaki et al., 2011; Wu et al., 2011), leading to renal structural destruction and renal functional injury in DN patients. Therefore, blocking the macrophage activation pathway may be of great significance in preventing chronic DN-related inflammation.

Recent studies have suggested that TLRs represent initiation pathways of macrophage activation during diabetic atherosclerosis (Hodgkinson et al., 2008). There are two pathways through which TLRs recruit downstream signaling molecules after identifying and binding with specific ligands, the MyD88-dependent signaling pathway and MyD88-independent signaling pathway. The pathways will eventually result in the transcriptional activation of NF-κB. NF-κB can regulate cytokines, cell adhesion molecules, inflammatory factors and other inflammation-related factors [such as monocyte chemotactic protein 1 (MCP-1), IL-6, IL-8, IL-18, and TNF-α] in DN once it is activated (Mudaliar et al., 2014; Ma et al., 2015). The role of endogenous, ligand-induced, TLR-mediated NF-κB activation has been studied in multiple nephropathy models (Bhattacharya et al., 2013; Ma et al., 2015). Of the studies of TLRs, research regarding TLR2 and TLR4 as well as their downstream signaling pathways has been the most extensive. Devaraj et al. (2011) used TLR4-/- mice to construct a streptozotocin (STZ)-induced diabetes model. Furthermore, they verified that knocking out TLR4 could suppress renal and abdominal macrophage activation and reduce inflammatory factor levels in the serum and macrophage numbers, thus decreasing renal inflammation (Devaraj et al., 2011). Lin et al. (2012) suggested that the TLR4-mediated signaling pathway in people with diabetes could lead to interstitial inflammation in renal tissue. In addition, Dasu et al. (2008) reported that high glucose (HG) levels can induce the upregulation of TLR2 and TLR4 expression *in vitro* in THP-1 human monocytes. Moreover, HG levels could activate the intracellular MyD88/IRAK-1/NF-κB signaling pathway. Furthermore, NF-κB p65 expression was downregulated, and inflammatory factor expression was also notably downregulated after silencing with a specific siRNA (Dasu et al., 2008). Hodgkinson et al. (2008) reported that AGEs can induce human and mouse macrophages to produce proinflammatory factors. However, the cytokine production stimulated by AGEs was remarkably reduced in mice with a functional mutation in the TLR4 gene (Hodgkinson et al., 2008). Consequently, scientists have correlated the TLR4 signaling pathway with the innate immune system and microvascular inflammatory response in people with diabetes, rendering this pathway a favorable target for anti-inflammatory treatments for DN.

In recent years, traditional Chinese medicine treatments have been extensively applied in DN patients. In our previous study, total glucosides of paeony (TGP) had a remarkable renal-protective effect and could inhibit the expression of TLR2 and TLR4 in the glomerulus and renal tubule-interstitial macrophages (Xu et al., 2014). Paeoniflorin (PF) is the major bioactive component of TGP, and the pharmacological effects of PF may be related to its inflammatory (Zhang et al., 2013), antioxidative (Zhang et al., 2013) and immunoregulatory (Wang et al., 2013) activities. Moreover, the mechanism by which PF suppresses HG-induced macrophage activation is partly achieved by regulating the TLR2-MyD88 pathway (Shao et al., 2016). This research was conducted to further determine whether PF can regulate TLR4 and its downstream signaling pathway to inhibit macrophage infiltration and activation in DN through *in vivo* and *in vitro* experiments. This study could provide new strategies for treating DN.

## Materials and Methods

### Materials

**FIGURE 1:**
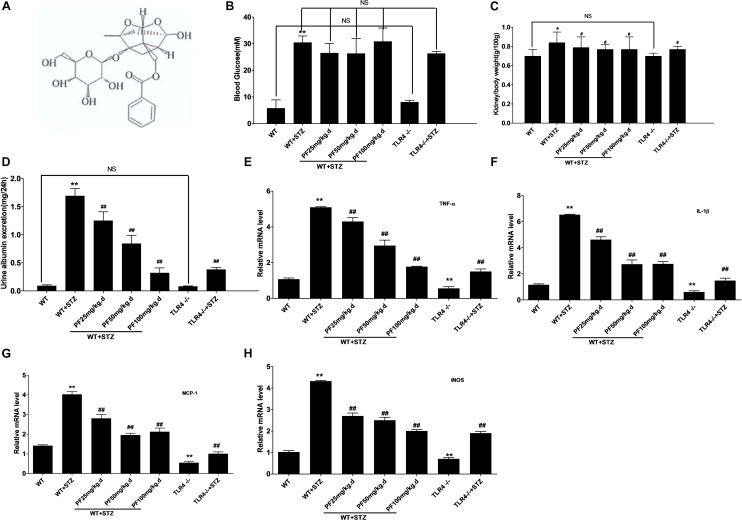
PF improved clinical symptoms and alleviated inflammation. **(A)** The chemical structure of paeoniflorin. **(B)** Blood glucose levels. **(C)** Kidney /body weight. **(D)** Urine albumin excretion. Data were detected and are presented as the mean ± SD of at least three independent experiments. *n* = 12 in each group. NS: not significant; ^∗^*p <* 0.05 and ^∗∗^*p* < 0.01 vs. WT; ^#^*p* < 0.05 and ^##^*p*<0.01 vs. WT+STZ. **(E–H)** Expression of iNOS, TNF-α, IL-1β, and MCP-1 in renal tissue. The results are represented as the mean ± SD of at least three repeated experiments. ^∗^*p* < 0.05 and ^∗∗^*p* < 0.01 vs. WT; ^#^*p* < 0.05 and ^##^*p* < 0.01 vs. WT+STZ; NS, not significant.

PF [C23H28O11, MW: 480.45, purity: 98.78% (HPLC), LD50: 9,530 mg/kg, Figure 1A] was obtained from Nanjing GOREN BIO Technology Co., Ltd. (Nanjing, China). PF was dissolved in saline to provide a stock solution. A microalbumin assay kit was acquired from Abcam Biotechnology (Abcam, Cambridge, United Kingdom). An immunohistochemistry kit (PV-9000) was purchased from Beijing Zhongshan Biotechnology Inc. (Zhongshan, China). Rabbit anti-TLR4, anti-MyD88, anti-Trif, and anti-iNOS antibodies were purchased from Abcam Biotechnology (Abcam, Cambridge, United Kingdom) and anti-p-IRF3, anti-NF-κB p65, and anti-NF-κB p-p65 antibodies were obtained from Cell Signaling Technology (CST Beverly, MA, United States). Rabbit anti-p-IRAK1 and anti-CD68 antibodies were obtained from Santa Cruz Biotechnology (Santa Cruz, CA, United States). Other materials for Western blotting (WB) were acquired from Amersham Life Science (Little Chalfont, United Kingdom). TRIzol was obtained from Invitrogen (Invitrogen, CA, United States), and the SYBR Green PCR Master Mix Kit was purchased from Bio-Rad (Bio-Rad, CA, United States). A cDNA synthesis kit was purchased from Promega (Promega, Madison, WI, United States). GAPDH and TNF-α primers were obtained from Shanghai Sangon Company (Shanghai, China).

### Mice

Wild-type (WT) C57BL/6J littermates and C57BL/10ScN mice (TLR4-/- mice) (males, 8–10 weeks of age) were purchased from the Model Animal Research Center of Nanjing University and housed individually in cages under standard conditions with a 12 h light-dark cycle and free access to food and water in a room with a temperature of 24°C and humidity of 60%. After 7 days of acclimation, the mice were provided with STZ daily (Sigma Chemical Co., St. Louis, MO, United States) in a citrate buffer (0.1 M, pH 4.5) at a dose of 50 mg/kg of body weight for 5 days to establish the diabetic model. PF was administered by intraperitoneal injection (i.p.) once a day for 12 weeks to the WT+STZ+PF group at doses of 25, 50, or 100 mg/kg. Other groups were administered equal amounts of normal saline. All animal experimental protocols were approved by the Ethics Committee of Animal Research of Anhui Medical University and executed according to the recommendations of the Laboratory Animal Care and Use guidelines.

### Physical and Biochemical Analyses

Body weight, kidney weight and blood glucose levels were measured. Twenty-four-hours urine samples were collected from the mice after 12 weeks in metabolic cages. The urinary albumin level was assayed using a mouse microalbumin enzyme-linked immunosorbent assay (ELISA) kit (Abcam Biotechnology, Cambridge, United Kingdom) according to the manufacturer’s instructions.

### Histological Analysis

For pathology and immunohistochemistry, 4% paraformaldehyde-fixed and paraffin-embedded fresh renal tissue sections were cut (2 μm thickness). After deparaffinization, the slides were stained with periodic acid-Schiff (PAS) reagent to identify kidney structures. The glomerular mesangial expansion index and the tubulointerstitial injury index were evaluated and graded using 10 randomly selected visual fields. The slides were treated with 3% hydrogen peroxide and then heated in a microwave to block the endogenous peroxidase activity and retrieve antigens, respectively. The tissue sections were blocked with normal horse serum for 30 min at 37°C and then incubated with a primary antibody. Anti-CD68 (Santa Cruz Biotechnology, CA, United States, 1:100), anti-TLR4 (Abcam Biotechnology, Cambridge, United Kingdom; 1:50), and anti-NF-κB p65 (CST, MA, United States, 1:100) antibodies were incubated overnight at 4°C with polyperoxidase-conjugated anti-mouse/rabbit IgG, followed by 3,3-diaminobenzidine (DAB, Sigma) and hematoxylin staining. The quantitation of CD68-positive cells, representing the recruitment of macrophages in the tissue section, was determined in 10 randomly selected high-power (400×) fields, and staining for TLR4 and NF-κB p65 was detected by ImagePro Plus Systems.

### Isolation and Culture of Bone Marrow-Derived Macrophages (BMDMs)

BMDMs were isolated from 6 to 8 weeks old male C57BL/10ScN mice (TLR4-/-) and their wild-type littermates (C57BL/6JWT) as described previously (Shao et al., 2016). We optimized the concentration and stimulation time of D-glucose and the PF concentration. D-mannitol was used as an osmolality control. The BMDMs were grouped as follows: (1) normal glucose concentration control group (LG), (2) normal glucose concentration +PF intervention group (LG+PF), (3) high-glucose stimulation group (HG), (4) PF intervention group (HG+PF), (5) normal glucose concentration TLR4 knockout group (TLR4-/-), (6) TLR4 knockout macrophages + high-glucose stimulation group (TLR4-/-+HG), and (7) TLR4 knockout macrophages + high-glucose stimulation + PF intervention group (TLR4-/-+HG+PF).

### Cell Viability Assay

Cell viability was detected using a CCK-8 kit (Vazyme, Nanjing, China) in accordance with the manufacturer’s instructions. Cell viability was detected by measuring the absorbance of the converted dye at 490 nm using a microplate reader (Synergy HT, Bio-Tek). The average optical density (OD) of control cells was set as 100% viability. The data were normalized and expressed as a percentage of the control.

### Cell Migration Assay

A total of 5 × 10^5^ cells were suspended and plated into the upper wells above the filter in a 24-well Boyden chamber (Corning 3422, United States). MCP-1 (PeproTech, Rocky Hill, United States; 20 ng/ml) was added to the lower wells for 24 h to act as an inductive agent. Then, the cells in the upper wells were removed by scraping, fixed using ice-cold methanol for 20 min, and stained with 0.1% crystal violet. Migrating cells were photographed using a light microscope at 200× magnification. Membranes were washed with phosphate-buffered saline (PBS), 200 μl of dye solubilization buffer (33% acetic acid) was added, and the OD was measured at 570 nm. Cell migration was arbitrarily set as 1 in each LG group.

### Flow Cytometry (FCM) Analyses

Antigens expressed on the cell surface, including of CD11c (BioLegend, San Diego, CA, United States), CD11b (BioLegend, San Diego, CA, United States) and F4/80 (BioLegend, San Diego, CA, United States), were detected by antibodies. BMDMs were blocked for nonspecific binding with anti-CD16/CD32 (BioLegend, San Diego, CA, United States), followed by PBS washing and centrifugation. The surface of the cells was stained with FITC-conjugated anti-mouse F4/80, PE-conjugated anti-mouse CD11c and APC-conjugated anti-human/mouse CD11b antibodies or isotype controls. The detailed steps were conducted as previously reported (Shao et al., 2016).

### Confocal Microscopy Analysis

Cells grown on the bottom of dishes were treated with PF for half an hour, exposed to HG levels for 24 h, and fixed with 4% paraformaldehyde for 15 min. Cell samples were blocked with 2% donkey serum albumin (EMD Millipore Corporation, United States) and stained with an anti-TLR4 antibody (Santa Cruz Biotechnology, CA, United States, 1:50) and anti-iNOS antibody (Abcam Biotechnology, Cambridge, United Kingdom, 1:50), followed by FITC-conjugated IgG (Santa Cruz Biotechnology, CA, United States, 1:200) and PE-conjugated IgG (Santa Cruz Biotechnology, CA, United States, 1:200). The nucleus was counterstained with 0.25 mg/ml 4-,6-diamidino-2-phenylindole (DAPI, Beyotime Institute of Biotechnology Jiangsu, China) for 5 min, the sections were mounted with antifade fluorescence mounting medium, and the macrophages were visualized using a Leica TCS SP5 laser confocal microscope (Leica, Germany) with the appropriate filters.

### RNA Extraction and Real-Time PCR

RNA was extracted from fresh renal tissue and BMDMs. TRIzol reagent (Invitrogen, CA, United States) was added to reverse-transcribed cDNA as part of the Reverse Transcription Kit (Invitrogen, CA, United States), and the cDNA in turn underwent RT-PCR using Power SYBR Green PCR Master Mix (Bio-Rad Laboratories, Hercules, CA, United States) and GAPDH primers (GAPDH primers: Forward primer 5′-ACCCCAGCAAGGACACTGAGCAAG-3′, Reverse primer 5′-GGCCCCTCCTGTTATTATGGGGGT-3′; Shanghai Sangon Company, Shanghai, China). The forward and reverse primers for the detected TNF-α RNA sequence were as follows: 5′-CCCTCCTGGCCAACGGCATG-3′ and 5′-TCGGGGCAGCCTTGTCCCTT-3′ (Shanghai Sangon Company, Shanghai, China); iNOS (MQP029793), IL-1β (MQP027422), and MCP-1 (MQP027672) primers were purchased from GeneCopoeia (Rockville, MD, United States). Finally, the relative expression levels of genes were analyzed by using the 2^-ΔΔCt^ method with values normalized to the expression of the reference gene GAPDH.

### Western Blot Analysis

Total protein was extracted from homogeneous renal samples that were lysed and combined with a phosphatase inhibitor cocktail in RIPA buffer (Beyotime Institute of Biotechnology Jiangsu, China). The protein concentration was detected with a Bio-Rad protein assay kit (Beyotime Institute of Biotechnology Jiangsu, China). After boiling, proteins were separated by 8–12% SDS-PAGE. The proteins were electroblotted onto a nitrocellulose membrane and incubated with one of the following specific primary antibodies: anti-TLR4, anti-MyD88, anti-Trif, anti-iNOS (Abcam Biotechnology, Cambridge, United Kingdom), anti-p-IRF3, anti-NF-κB p65, anti-NF-κB p-p65 (CST, Beverly, MA, United States) or anti-p-IRAK1 (Santa Cruz Biotechnology, CA, United States). A horseradish peroxidase-labeled secondary antibody (Wuhan Sanying Biotechnology Inc., Wuhan, China) was added after the membrane was washed. Finally, the bound secondary antibody was detected by enhanced chemiluminescence (Amersham Life Science, Little Chalfont, United Kingdom), and the protein content was quantified and analyzed using a Leica Q500IW image analysis system.

### ELISA Detection

Culture medium was removed at the indicated time points. Soluble cytokines, including MCP-1 (RIBIO TECH, Beijing, China), IL-1β (R&D Systems, United States) and TNF-α (R&D Systems, United States), were assayed by commercially available ELISA detection kits according to the manufacturer’s instructions.

### Statistical Analysis

Data were analyzed with SPSS 16.0. Normally distributed data were expressed as the mean ± SD, and all the compared data were tested by one-way ANOVA. The differences among groups were tested by the least significant difference (LSD) test and the Levene method for homogeneity test of variance, in which a *P*-value less than 0.05 was considered significant.

## Results

### PF Could Improve Clinical Symptoms and Alleviate Inflammation

First, STZ-induced diabetic mice were intraperitoneally injected with PF. As shown in Figure 1B, the STZ injection group showed remarkably higher blood glucose levels than the WT and TLR4-/- groups. However, PF treatment could not evidently reduce the blood glucose levels, which was a result that differed from the results of Fu et al. (2009) in diabetic rats. Compared with nondiabetic WT and TLR4-/- mice, STZ-injected mice had an elevated kidney/body weight ratio and increased urine albumin excretion (Figure 1C,D). In contrast, PF treatment could reduce the kidney/body weight ratio and urine albumin excretion of STZ-induced diabetic mice (WT-STZ-PF treatment group). However, the levels in these mice were still higher than those in WT and TLR4-/- mice.

Subsequently, the mRNA expression of inflammatory factors and iNOS in the kidneys was observed, and the results are displayed in Figure 1E–H. As can be seen, PF treatment and knocking out TLR4 could remarkably reduce the expression levels of TNF-a, IL-1B, MCP-1, and iNOS in diabetic mouse kidneys.

### PF Treatment Could Alleviate the Pathological Manifestations of DN

**FIGURE 2:**
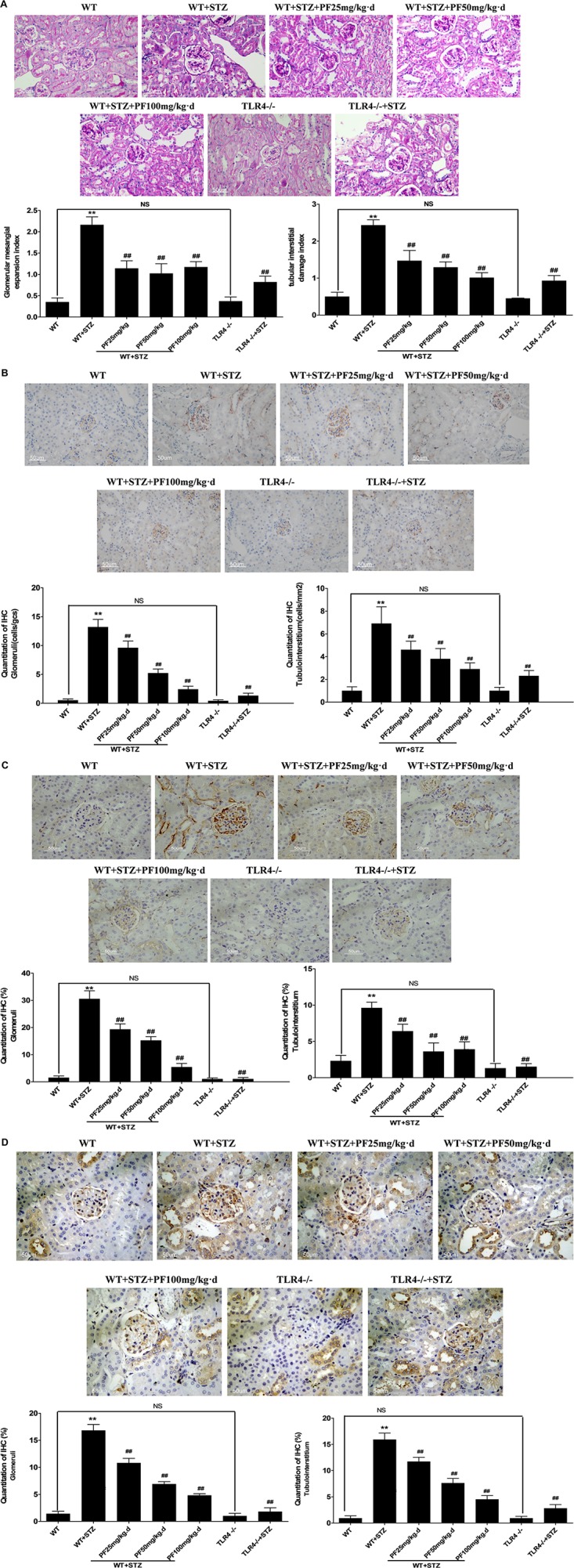
PF treatment could alleviate the pathological manifestations of DN. **(A)** Histological observations of kidney sections stained with PAS. **(B)** Quantitation of CD68-positive cells, representing the recruitment of macrophages in the tissue. Macrophages were counted in randomly selected high-power (400×) fields. **(C,D)** Immunohistochemistry results evaluated as the average positive stained area percentage by ImagePro Plus Systems. The results are represented as the mean ± SD; *n* = 10 in each group. ^∗^*p* < 0.05 and ^∗∗^*p* < 0.01 vs. WT; ^#^*p* < 0.05 and ^##^*p* < 0.01 vs. WT+STZ; NS, not significant.

Figure 2A shows the histological observations of kidney sections stained with PAS. In the model group, the kidneys increased in volume, and the glomerular mesangial cells, renal tubular epithelial cells and basement membrane were stained dark red. The glomerular mesangial expansion index and tubulointerstitial damage index were significantly increased, which was inconsistent with the expected pathology of early-stage DN. Within the PAS-positive range of the PF intervention and TLR4 gene knockout groups, the glomerular mesangial expansion index and tubulointerstitial damage index were significantly decreased. Related studies have clearly demonstrated that the mesangial dilatation index and tubulointerstitial damage index are closely related to renal function (Moriya et al., 2008). These data indicated that PF could noticeably prevent the progression of DN.

Macrophage infiltration is a major clinical feature of DN. CD68, a parameter representing renal macrophage accumulation (Klessens et al., 2017), was occasionally observed in the kidneys from WT and TLR4-/- mice. In comparison, CD68-positive macrophage infiltration was remarkably increased in WT+STZ mice. Immunostaining indicated a significant reduction in positive CD68 expression in the kidneys from PF-treated and TLR4-/-+STZ mice compared with those from WT+STZ mice. This finding verified the effects of PF on macrophage accumulation and infiltration, showing that PF had the same effect as knocking out TLR4 (Figure 2B).

Subsequently, the expression of TLR4 and NF-κB p65 was examined in mouse renal tissue since the relationship between TLR4 and the proinflammatory state in renal tissue is associated with diabetes. As was expected, positive TLR4 immunohistochemical staining was barely noted in the kidneys from TLR4-/- and TLR4-/-+STZ mice. Compared with WT mice, WT+STZ mice illustrated typical overexpression of TLR4 (Figure 2C). The intensity of the TLR4 immunostaining was strikingly decreased after PF treatment in a dose-dependent manner (Figure 2C). This result showed that PF treatment could downregulate TLR4 expression in a diabetic model. In addition, NF-κB p65 was also remarkably overexpressed in the nucleus and cytoplasm of glomerular cells and renal tubular cells in WT+STZ mice compared with the low expression in those cells in WT mice. However, NF-κB p65 expression was downregulated after PF treatment or knocking out TLR4 (Figure 2D).

### Effects of PF on the Expression of TLR4 and Downstream Signaling Pathway Molecules in Mice From Different Groups

**FIGURE 3:**
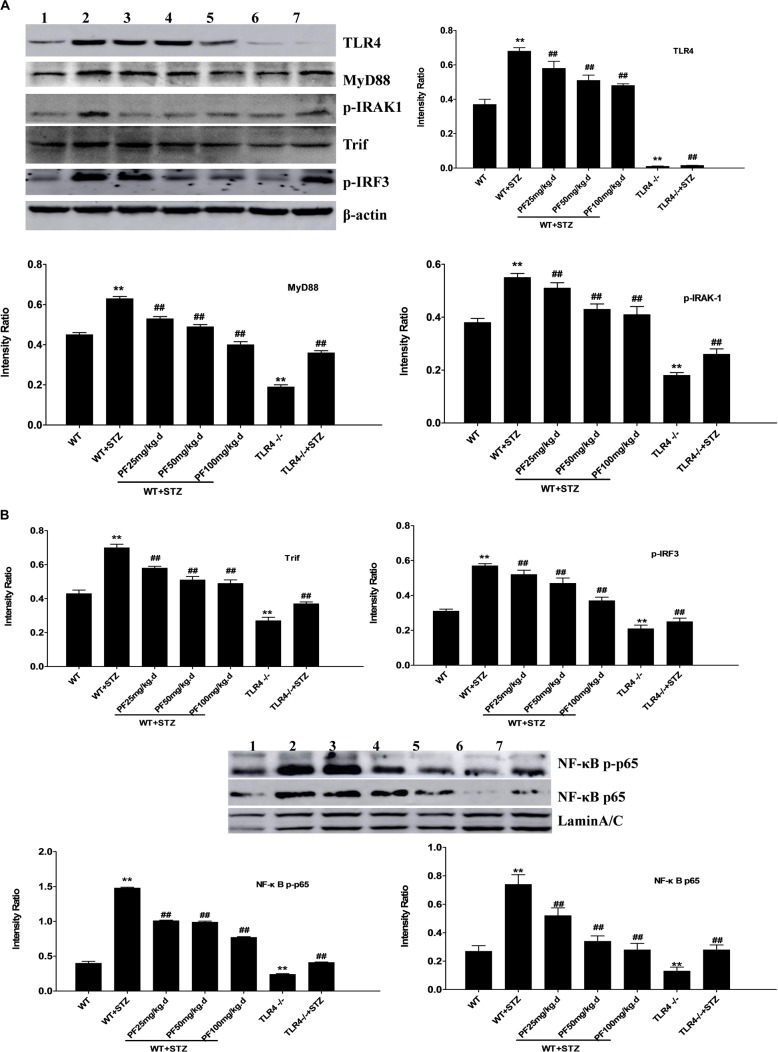
Effects of PF on the expression of TLR4 and downstream signaling pathway molecules in mice from different groups. **(A,B)** Renal tissue lysates were analyzed by Western blotting to measure the expression of TLR4 and molecules in the downstream signaling pathway, including MyD88, p-IRAK1, Trif, p-IRF3, NF-κB p-p65, and NF-κB p65. All data are expressed as the mean ± SD from at least three independent experiments. ^∗^*p* < 0.05 and ^∗∗^*p* < 0.01 vs. WT; ^#^*p* < 0.05 and ^##^*p* < 0.01 vs. WT+STZ; NS, not significant. Lanes: 1, WT; 2, WT+STZ; 3, WT+STZ+PF 25 mg/kg; 4, WT+STZ+PF 50 mg/kg; 5, WT+STZ+PF 100 mg/kg; 6, TLR4-/-; and 7, TLR4-/-+STZ.

The expression of TLR4 in different groups as well as that of downstream signaling molecule proteins was further detected using Western blot analysis. The results indicated markedly upregulated expression of TLR4, MyD88, p-IRAK1, Trif, p-IRF3, NF-κB p-p65, and NF-κB p65 in the WT+STZ group compared with the WT or TLR4-/- group. In comparison, the expression of the abovementioned proteins was significantly downregulated after PF treatment or knocking out TLR4 (Figure 3A,B).

### Cellular Experimental Conditions

**FIGURE 4:**
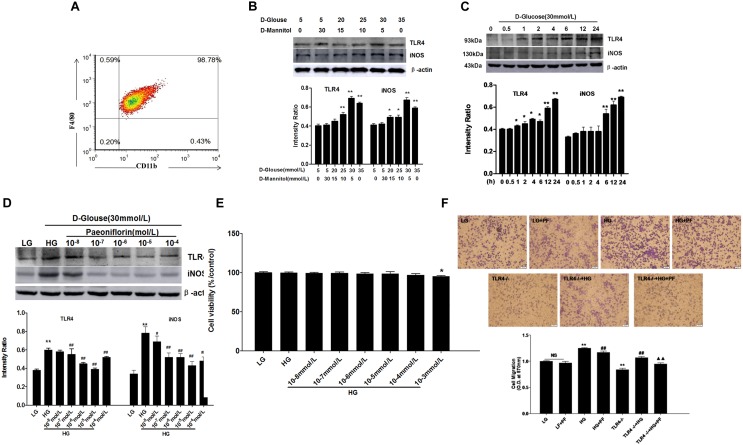
Cellular experimental conditions. The optimal concentration and stimulation time of D-glucose and the optimal PF concentration were determined first. **(A)** Identification of BMDMs. **(B)** Effects of different high concentrations of glucose on TLR4 expression and BMDM activation. **(C)** Effects of high-glucose stimulation on TLR4 and BMDM activation at different time points. **(D)** Effects of various PF concentrations on TLR4 expression and BMDM activation. **(E)** Cell viability detected using a CCK-8-based assay. **(F)** Cell migration detected by the Transwell method. Values are expressed as the mean ± SD of at least three independent experiments. NS: not significant; ^∗^*P <* 0.05 and ^∗∗^*P <* 0.01 vs. the LG group; ^#^*p* < 0.05 and ^##^*p* < 0.01 vs. the HG group; ^▲^*P <* 0.05, ^▲▲^*P <* 0.01 vs. the TLR4-/-+HG group.

Experimental conditions were optimized at the initiation of the cellular experiment. In Figure 4A, F4/80 and CD11b double labeling was conducted with isolated and cultured mouse BMDMs. The results suggested that the macrophage purity was as high as 99.61%. Different concentrations of dextrose were used to stimulate the macrophages. Moreover, mannitol was added to the cell medium to eliminate the influence of osmotic pressure. As seen in Figure 4B, TLR4 and iNOS protein expression was highest at the dextrose concentration of 30 mmol/l. In Figure 4C, TLR4 expression began to increase 1 h after experiment initiation and peaked 24 h later. Similarly, iNOS protein expression began to increase 6 h after experiment initiation and peaked at 24 h. In Figure 4D, different concentrations of PF were selected to treat HG-induced macrophages, and the results suggested that PF at a concentration of 10^-5^ mol/L exerted the most prominent inhibitory effect on TLR4 and iNOS protein expression in the HG-stimulated macrophages.

### Effects of PF on BMDM Cell Viability and Migration

Under HG induction conditions, different concentrations of PF (ranging from 10^-8^ to 10^-3^ mol/L) were used to treat macrophages. As shown in Figure 4E, no concentration of PF other than 10^-3^ mol/L showed an influence on cell proliferation.

A Transwell experiment was selected as a chemotaxis experiment. All groups in the experiment were tested for MCP-1-induced chemotaxis, and HG induction, PF intervention and TLR4 gene knockout were tested based on the experimental design. As can be seen in Figure 4F, HG treatment promoted macrophage migration, while PF intervention and knocking out TLR4 could act against the chemotaxis induced by MCP-1 in macrophages. Moreover, PF could further inhibit HG-induced macrophage migration after the TLR4 gene was knocked out.

### PF Decreased HG-Induced BMDM Differentiation Toward a Proinflammatory Phenotype

**FIGURE 5:**
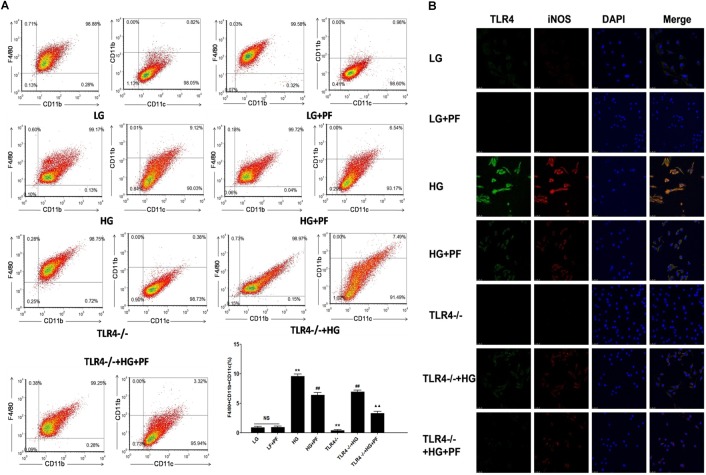
PF decreased HG-induced BMDM differentiation toward a proinflammatory phenotype. **(A)** BMDM activation analysis showing the effects of PF on the expression of macrophage surface markers in HG-induced BMDMs. F4/80 and CD11b are surface markers of macrophages, and CD11c is considered a marker of M1-type macrophages. The markers were assessed by flow cytometry. The results are expressed as the mean ± SD of at least three independent experiments. ^∗∗^*P* < 0.01 vs. the LG group; ^##^*P* < 0.01 vs. the HG group; ^▲▲^*P* < 0.01 vs. the TLR4-/-+HG group; NS: not significant. **(B)** The coexpression of TLR4 and iNOS in HG-induced BMDMs assessed by confocal microscopy. The effects of PF on the expression of TLR4 signaling pathway molecules and activation of macrophages in HG-induced BMDMs were evaluated by confocal microscopy analysis.

Positive CD11c expression was considered the marker of HG-induced BMDM differentiation toward the proinflammatory macrophage phenotype. As can be seen in Figure 5A, HG stimulation markedly increased the CD11c-positive cell percentage, while PF intervention and knocking out TLR4 could distinctly reduce the CD11c-positive proportion. Furthermore, compared with the TLR4-/-+HG group, the PF+TLR4-/-+HG group had a further decrease in the proportion of CD11c-positive cells. The synergistic relationship between TLR4 and the macrophage inflammatory phenotype was observed using laser confocal microscopy. In Figure 5B, compared with the LG group, the HG group had notably enhanced TLR (green) and iNOS (red) fluorescence signals, while PF intervention and knocking out TLR4 could inhibit the enhancement of the fluorescence signals. This finding suggested that HG levels could activate TLR4 and iNOS expression in the macrophage cytoplasm, which could be prevented by PF.

### PF Could Suppress iNOS and Inflammatory Factor Levels in HG-Induced Macrophages

**FIGURE 6:**
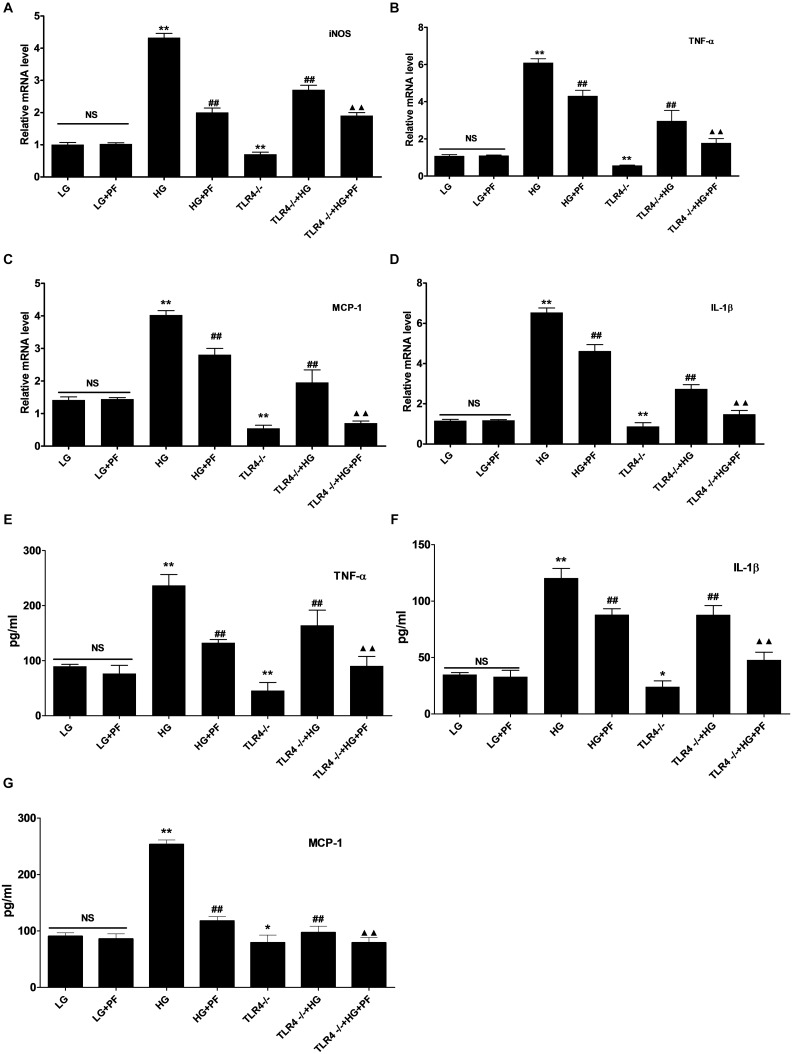
PF could suppress iNOS and inflammatory factor levels in HG-induced macrophages. **(A–D)** Effects of PF treatment and TLR4 deficiency on HG-stimulated iNOS and inflammatory cytokine expression. BMDMs were treated with HG stimulation for 24 h, and mRNA levels were detected by qRT-PCR. **(E–G)** Detection of the levels of TNF-α, IL-1β, and MCP-1 secreted by HG-stimulated BMDMs. Conditioned medium was removed, and ELISAs were used to detect soluble TNF-α, IL-1β and MCP-1 peptides. ^∗^*P* < 0.05 and **^∗∗^***P* < 0.01 vs. the LG group; **^##^***P* < 0.01 vs. the HG group; ^▲▲^*P* < 0.01 vs. the TLR4-/-+HG group; NS: not significant.

The effect of HG on macrophage activation was observed using PCR, and the secretion of inflammatory factors into the cell medium was determined by ELISA. Compared with the LG group, the HG group showed notably upregulated iNOS, TNF-α, IL-1β, and MCP-1 mRNA expression levels. At the same time, the TNF-α, IL-1β, and MCP-1 levels in the cell medium were also notably elevated. PF intervention and knocking out TLR4 could distinctly suppress iNOS and inflammatory factor transcription, which reduced the secretion of inflammatory factors into the medium. Compared with the TLR4-/-+HG group, the PF+TLR4-/-+HG group had further decreases in the transcription and secretion of iNOS and inflammatory factors (Figure 6A–G).

### PF Inhibited the Activation of TLR4 and Its Downstream Signaling Pathway in HG-Induced BMDMs

**FIGURE 7:**
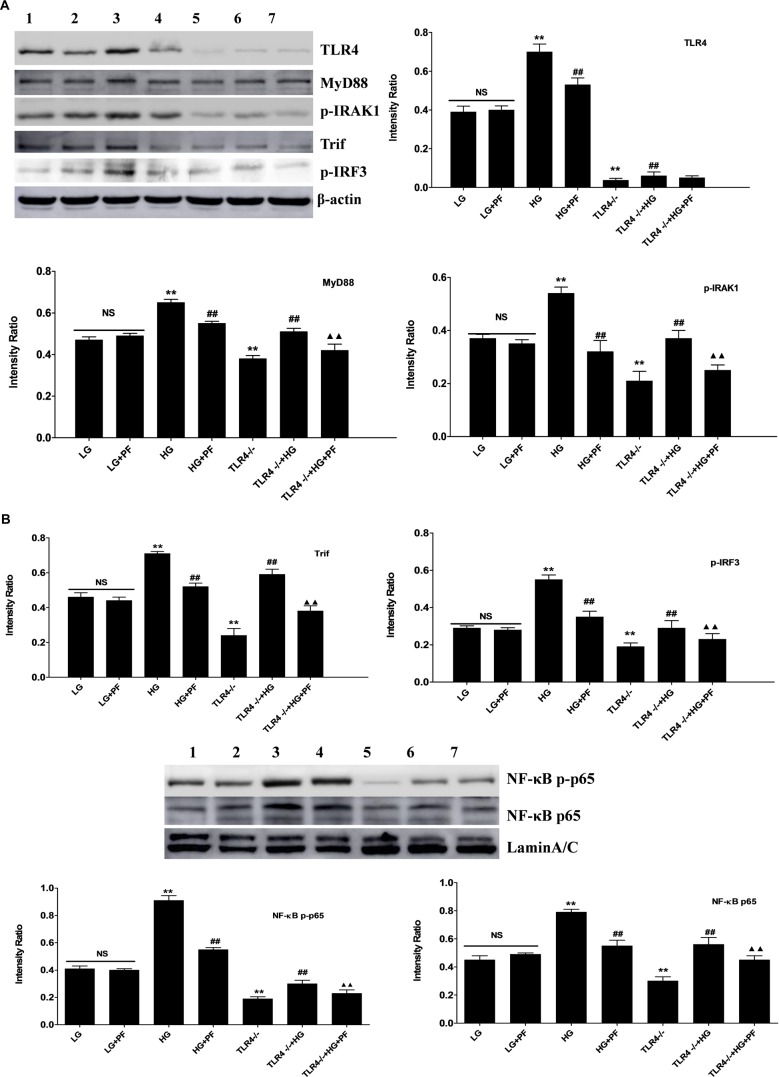
PF inhibited the activation of TLR4 and its downstream signaling pathway in HG-induced BMDMs. **(A)** Densitometric analyses of the protein levels of TLR4, MyD88, p-IRAK1, Trif, p-IRF3 and IRF3 in HG-treated BMDMs. **(B)** Densitometric analyses of the protein levels of NF-κB p-p65, and NF-κB p65 in HG-treated BMDMs. All data were normalized to the β-actin level and are expressed as the mean ± SD from at least three independent experiments. NS: not significant; ^∗^*P <* 0.05 and ^∗∗^*P <* 0.01 vs. the LG group; ^#^*p* < 0.05 and ^##^*p* < 0.01 vs. the HG group; ^▲^*P <* 0.05 and ^▲▲^*P <* 0.01 vs. the TLR4-/-+HG group. Lanes: 1, LG; 2, LG+PF; 3, HG; 4, HG+PF; 5, TLR4-/-; 6, TLR4-/-+HG; and 7, TLR4-/-+HG+PF.

Western blot analysis showed that the levels of TLR4, MyD88, p-IRAK1, Trif, p-IRF3, NF-κB p-p65, and NF-κB p65 were distinctly higher in the HG group than in the LG group. Furthermore, the PF intervention and TLR4-/-+HG groups showed significantly decreased expression. In addition, PF could further inhibit the HG-induced overexpression of downstream signaling molecules in the TLR4 pathway in TLR4-/- BMDMs (Figure 7A,B).

## Discussion

DN is a long-term complication of diabetes, and preventing its progression has remained a challenge. The accumulated evidence indicates that albuminuria is a leading risk factor for DN progression (Bakris, 2008; Amor et al., 2013). Renal hypertrophy, which is defined as an increased kidney/body weight ratio, is one of the major manifestations of renal involvement during incipient DN (Bakris, 2008). The present study demonstrated that PF intervention and knocking out TLR4 can decrease the urine albumin level and ameliorate the kidney/body weight ratio, thus obstructing the progression of DN. Blood glucose control is critical in the treatment of DN. However, the results of our experiment, which are different from the results reported in Fu et al. (2009) for diabetic rats treated with a dose of 5–20 mg/kg, demonstrate that PF has no effect on the blood glucose level. Another study showed that PF can obviously reduce the plasma glucose level by 1 mg/kg, but this effect became less pronounced as the dose increased (Hsu et al., 1997). Therefore, the remission in experimental DN mice caused by the hypoglycemic effect can be debated. In addition, the mechanisms by which PF intervention and knocking out TLR4 delayed DN progression were further investigated.

Inflammation is an important pathological process in DN (Wada and Makino, 2013), and macrophages are one of the important inflammatory cell types. Macrophage infiltration occurs during the early stage of DN and is one of the characteristic manifestations of DN (Tesch, 2010; You et al., 2013; Klessens et al., 2017). Nguyen et al. (2006) discovered from renal biopsies of 20 DN patients collected over 5 years that macrophage infiltration into the renal tissue is positively correlated with albuminuria, the serum creatinine level and the pulmonary interstitial fibrosis severity. This result suggested that macrophage infiltration into the renal tissue is closely correlated with renal functional decline (Nguyen et al., 2006). Macrophages could be observed in CD68-labeled renal tissue samples in our research, verifying the overexpression of CD68 in STZ-induced DN mouse renal tissue. In addition, PF intervention and knocking out TLR4 could notably downregulate CD68 expression, confirming the role of PF in reducing macrophage infiltration into the renal tissue.

The key to macrophage infiltration into the kidneys involves the passage of monocytes through the renal vascular endothelium to enter the renal parenchyma. This passage is related to MCP-1 and osteopontin. Research has indicated that HG levels can stimulate macrophage migration (Cheng et al., 2015). Moreover, in a renal tubular epithelial cell experiment *in vitro*, the application of HG and AGE stimulation elevated MCP-1 expression, while using HG and angiotensin II could increase osteopontin expression (Sasai et al., 2012). Similar results have been obtained in our research. MCP-1 was used as the initial chemotactic factor, and cells in all the experimental groups underwent chemotactic migration in response to stimulation with a HG level. However, compared with the control group incubated with a normal glucose concentration, the HG stimulation group showed markedly elevated macrophage migration, which could be inhibited by PF intervention and knocking out TLR4.

Research has discovered that, in different microenvironments, macrophages can be activated into different histological subtypes: classically activated (M1) and alternatively activated (M2). Current studies suggest that M1-type cells mainly mediate renal inflammation and fibrosis, while M2-type cells have the functions of tissue repair and fibrosis inhibition (Sik et al., 2011). M1-type macrophage accumulation in the kidneys is an important feature of DN (Sato et al., 2008). The more recognized M1 markers are iNOS, TNF-, and IL-1. In the early stage of injury, M1-type macrophages can induce the production of reactive oxygen species, promote the synthesis of NO, and release various inflammatory factors, such as iNOS, TNF-α, and IL-1β, resulting in damage to glomerular intrinsic cells and the basement membrane, thereby causing proteinuria and glomerular inflammation (Kianoush et al., 2017). Similar results can be observed in HG-stimulated BMDMs. Specifically, PF intervention and knocking out TLR4 can distinctly reduce inflammatory phenotype development in macrophages, inhibit HG-stimulated macrophage inflammatory factor and iNOS transcription, and decrease inflammatory factor levels in cell culture supernatants. In addition, we found through the synergistic expression of iNOS and TLR4 in macrophages under HG stimulation conditions that HG-stimulated macrophage activation is closely correlated with TLR4 expression.

Macrophage infiltration and activation in diabetic renal tissue are closely correlated with TLRs (Jialal and Kaur, 2012). TLRs are expressed in renal tubular epithelial cells, mesangial cells, podocytes and monocytes/macrophages (Vasavada and Agarwal, 2005). Recently, research on the roles of TLR family members, especially TLR2 and TLR4, in DN inflammation has attracted extensive attention. TLR2/4 can identify multiple ligands in the diabetic environment, activate NF-κB and subsequently activate the inflammatory cascade (Schmid et al., 2006; Navarro-Gonzalez et al., 2011). Our immunohistochemical results have also provided evidence for TLR4 and NF-κB p65 activation in DN renal tissue. Furthermore, we also analyzed TLR4 and its downstream signaling pathway using Western blotting. We observed that TLR4 and its downstream signaling pathways are notably activated in HG-stimulated macrophages in renal tissue. This activation manifests as upregulated expression of the downstream MyD88-dependent and MyD88-independent pathways, accompanied by increased transcription of inflammatory factors and iNOS. According to our experimental results, knocking out TLR4 can downregulate the activity of the TLR4 signaling pathway and distinctly reduce CD68 expression, which represents macrophage infiltration. In addition, there was decreased expression of inflammatory factors and iNOS, which suggested macrophage activation. These results revealed that macrophage infiltration and activation in DN renal tissue can be regulated by TLR4 and its downstream signaling pathway.

PF, a main bioactive component of TGP, can be extracted from the dried peeled root of *Paeonia lactiflora* Pall. Some reports have supported a positive role for PF in preventing DN (Hsu et al., 1997; Fu et al., 2009). Zhang et al. (2013) provided reliable evidence that PF attenuates AGE-induced oxidative damage and inflammation in mesangial cells. Another report found that PF inhibits autophagy at least partly by inhibiting the RAGE/mTOR pathway (Chen et al., 2017). On the other hand, there is compelling evidence indicating that the targeted inhibition of abnormal TLR4 overexpression is valuable for alleviating DN progression (Fu et al., 2009; Devaraj et al., 2011; Shao et al., 2016). Therefore, this study was conducted to explore whether the beneficial effect of PF is mediated in part by changing the expression of TLR4 signaling pathway molecules. In the present study, PF intervention had the same therapeutic effect as knocking out TLR4. Specifically, we observed that PF intervention can decrease albuminuria and alleviate renal hypertrophy in WT+STZ mice. In addition, we noted that PF can block NF-κB p65 activation and macrophage recruitment as well as suppress inflammatory cytokines and chemokines. In addition, the present study showed that the increased TLR4 levels in DN can be suppressed by PF treatment.

The *in vitro* experiment showed that PF can further suppress the expression of the MyD88-dependent and MyD88-independent downstream signaling pathways, activation of macrophages and release of inflammatory factors in the TLR4 knockout group. Therefore, the PF-mediated regulation of inflammation does not depend entirely on the TLR4 signaling pathway in DN. Inflammation is related to many signaling pathways in the early stage of DN. PF has been proven to inhibit the production of inflammatory factors by regulating some signaling pathways, and TLR signaling pathways are the most frequently studied. In support of our data, our previous animal studies suggested that the protective effect of TGP on DN is associated with the blockade of TLR2 and TLR4 activation in tubular cells, glomerular cells, and macrophages. Zhang et al. (2014) recently indicated that PF can abrogate BPS-induced sepsis by decreasing the activity of TLR2 signaling pathways. We speculate, based on these observations, that PF can improve the inflammatory status in DN through inhibiting the TLR4 pathway. Moreover, the anti-inflammatory mechanism of PF is also associated with other signaling pathways (Shao et al., 2016; Li et al., 2018).

## Conclusion

In summary, our findings have suggested that TLR4 activation can initiate macrophage infiltration and M1 polarization, resulting in the release of inflammatory cytokines and chemokines. In turn, these effects on macrophages will exacerbate inflammation and ultimately aggravate DN. PF treatment can ameliorate the clinical symptoms, delay the onset and alleviate the severity of DN. Furthermore, the therapeutic effect of PF is associated with apparently reduced macrophage infiltration, reduced TLR4 signaling pathway activation, and the release of inflammatory cytokines.

## Ethics Statement

All animal experimental protocols were approved by Biomedical Ethics Committee of Anhui Medical University and executed, according to the recommendations of Laboratory Animal Care and Use.

## Author Contributions

Y-gW and KW conceived and designed the experiments. Y-xS and QG performed the experiments. Y-xS and X-MQ analyzed the data, drafted and edited the manuscript. All authors approved the final version to be published.

## Conflict of Interest Statement

The authors declare that the research was conducted in the absence of any commercial or financial relationships that could be construed as a potential conflict of interest.

## Abbreviations

BMDM: bone marrow-derived macrophage
HG: high glucose
PF: paeoniflorin
STZ: streptozotocin
TLR4: toll-like receptor 4
